# The risk factors of Urethrocutaneous fistula after hypospadias surgery in the youth population

**DOI:** 10.1186/s12894-018-0366-z

**Published:** 2018-07-24

**Authors:** Xujun Sheng, Ding Xu, Yu Wu, Yongjiang Yu, Jianhua Chen, Jun Qi

**Affiliations:** Department of Urology, School of Medicine, Xinhua Hospital, Shanghai Jiao Tong University, 1665 Kongjiang Rd, XinHua Hospital, Shanghai, 200092 China

**Keywords:** Hypospadias, Urethrocutaneous fistula, Risk factor, Urethral defect length, Operation history

## Abstract

**Background:**

The current research aims to evaluate the risk factors of urethrocutaneous fistula after hypospadias surgery among the youth in China.

**Methods:**

One hundred twenty hypospadias patients were enrolled in our study. All of them were defined as Tanner 4 or 5. The information collected from the participants include age, urethral operation history, urinary comorbidities before operation, urine test before operation, body temperature before and after operation, type of surgical repair, chordee degree, urethral defect length and whether received vesicostomy after surgery or not. Independent t test, chi-square test and multivariate logistic regression were performed to evaluate the risk factor of urethrocutaneous fistula.

**Results:**

Among the enrolled patients, 39 patients (32.5%) developed urethrocutaneous fistula after hypospadias repair. Our result showed significant association between the group with urethrocutaneous fistula and the group without urethrocutaneous fistula with respect to age, pyuria before operation, urethral defect length and the urethral operation history. The following logistic regression showed that urethral defect length and the urethral operation history were the risk factors of urethrocutaneous fistula.

**Conclusions:**

Urethral defect length and urethral operation history should be taken into consideration before undergoing hypospadias surgery since our study discovered that the risk of developing urethrocutaneous fistula after hypospadias repair is associated with urethral defect length and urethral operation history. Age, surgical procedure, type of surgical repair, chordee degree and other factors were not obviously related to the development of urethrocutaneous fistula.

**Electronic supplementary material:**

The online version of this article (10.1186/s12894-018-0366-z) contains supplementary material, which is available to authorized users.

## Background

Hypospadias, in which the urethral opening occurs on the ventral side of the penis, is the most common congenital condition of the penis. The incidence of hypospadias ranged from very low rate of 0.6/10,000 births (Malaysia) to extremely high rate of 464/10,000 births (Denmark). Low prevalence was also reported from China (0.7–4.5/10,000). However, the increasing trend in the prevalence has been shown [1]. Meanwhile, Hypospadias surgery has been in continuous evolution for many years with steadily improving reported results [2]. However, The results of hypospadias surgery are still frequently unfavourable with reported complication rate as high as 50% or above [3, 4]. The most common complications following hypospadias surgery accompany with urethrocutaneous fistula, meatal stenosis, urethral stricture, urethral diverticulum, glans dehiscence, breakdown, and cosmetic unfavorable outcome requiring redo-surgery [5]. Urethrocutaneous fistula, followed by hypospadias reconstruction, is one of the most common complications. Post-surgery fistula in children could occur as the result of one or more factors, such as meatal stenosis, urorethral stricture, hematoma, infection, poor surgical technique, etc. [6]. However, a lot of patients, especially those in the developing countries, go to outpatient department for treatment when they are grown up, possibly restrict to economic factors, unsuccessful surgery history or other reasons. There are a few other reports specifically concerning the hypospadias surgical outcomes among the youth population. Some studies reported higher complication rate occurs among adults than children using the same techniques [7], but another study argue against it [8]. Moreover, up to now, the risk factors of Urethrocutaneous fistula after hypospadias surgery in the youth are still unknown. The aim of this study is to evaluate these risk factors of Urethrocutaneous fistula after hypospadias surgery among youth population in China.

## Methods

The retrospective study involved 120 patients who were treated in our department suffering from hypospadias from Jan 2002 to Dec 2013. Those who were defined as Tanner 4 or 5 and were followed up by their surgeons for evaluating the effect of operation from 6 months to 2 years (11.75 ± 3.89 months) were primarily focused in our study. This study was approved by the Ethics Committee of Xin Hua Hospital Affiliated to Shanghai Jiao Tong University School of Medicine [2014–007]. The information of the participants were collected on age, urethral operation history, urinary comorbidities before operation, infection before and after operation, type of surgical repair, chordee degree, urethral defect length and whether received vesicostomy or not after surgery. Urethral operation history was divided into 2 groups: one represented none; two represented the history of at least one urethral operations before the current surgery. Pyuria before surgery was defined as white blood cell> 5/HP in urine test. Infection after surgery was defined as body temperature > 38 °C. Chordee was quantified preoperatively by Horton test divided into mild (0–20°), moderate (30–40°) and severe (> 50°) [9]. Urethral defect length was measured after correction of chordee during the operation. The choice of the procedure was based on surgeon’s experience and the characteristics of the urethral plate regardless of the meatal location. The hypospadias repairs can be classified into single-stage procedures and two-stage urethral plate substitution procedure (Bracka’s repair). The single-stage procedures are (a) urethral plate tubularization (glanular approximation and Snodgrass repair) and (b) urethral plate augmentation (onlay lap and Snodgraft repair) [10]. All operations were conducted by four experienced urologists.

Statistical Package for Social Science(SPSS), version 13.0 was used for statistical analysis. The data was presented in the form of mean ± SD. Independent t-test was used to calculate the numerical parameters of two groups having significant difference when the parameter was consistent with normal distribution and Mann-Whitney U test was used when the parameter was consistent with nonparameter distribution. Chi-square test was analyzed in categorical parameters. Binary logistic regression was used in multivariate analysis to find the risk factors of urethrocutaneous fistula after hypospadias surgery in adults in China. For all statistical tests, a *P*-value < 0.05 was considered to be statistically significant.

## Results

The median age of enrolled patients at surgery was 13.50 (11–42) years old. Thirty-nine patients developed urethrocutaneous fistula after surgical repair, which meant the complication rate was 32.5%. Five patients’ hypospadias could not be completely repaired. Three patients met urethral stenosis. Seven patients met fistula associated with meatal stenosis while 1 patient met fistula associated with diverticulum. Among the enrolled patients, 62 patients (51.67%) received at least one hypospadias repair surgery before. The mean urethral defect length was 4.11 ± 2.70 cm. Sixteen patients (13.33%) received two-stage procedure and 18 patients (15%) received bladder stoma after the opreation. Table 1 shows that the age, the pyuria before operation, urethral defect length and urethral operation history had significant differences between the group with urethrocutaneous fistula and the group without urethrocutaneous fistula. The chordee showed no signicfant difference between two group from Table 2. With the following multivariate logistic regression, we found that two parameters, urethral defect length (OR1.215, 95%CI: 1.009–1.464) and urethral operation history (OR 2.469, 95% CI: 1.021–5.974) were the two independent risk factors of urethrocutaneous fistula. The correlation among all the parameters was summarized in Table 3.

**Table 1:** The comparison of clinical parameters between the group with fistula and the group without fistula

	Without fistula (*n* = 81)	With fistula (*n* = 39)	*P* value
age	14.46 ± 3.80	16.82 ± 5.99	0.010*€
Pyuria before surgery	2.47% (2/81)	12.82% (5/39)	0.023*#
Urinary comorbidities before surgery	14.81% (12/81)	28.21% (11/39)	0.081#
Urethral defect length	3.75 ± 3.04 cm	4.86 ± 1.58 cm	0.034*€
Urethral operation history	43.21% (35/81)	69.23%(27/39)	0.008*#
urethral plate tubularization	59.26%(48/81)	46.15% (18/39)	0.177#
penis vascular pedicle flap	39.51% (32/81)	56.41% (22/39)	0.081#
free flap from oral cavity	6.17% (5/81)	0 (0/39)	0.113#
Two-stage surgical procedure	14.81% (12/81)	10.26% (4/39)	0.491#
bladder stoma	12.35% (10/81)	20.51% (8/39)	0.241#
Fever after surgery	9.88% (8/81)	23.08% (9/39)	0.052#

*P < 0.05 #chi-square test €independent t test

**Table 2:** The comparison of penile chordee between two groups

		Without fistula (*n* = 81)	With fistula (*n* = 39)	*P* value
Penile chordee	Mild (0–20°)	23	13	0.737
	Moderate (30–40°)	35	14	
	Severe (> 50°)	23	12	

*P* < 0.05

**Table 3:** The correlation among all the clinical parameters

		Age	Pyuria before surgery	Urinary comorbidities before surgery	Urethral defect length	Penile chordee	Urethral plate tubularization	Free flap from oral cavity	Penis vascular pedicle flap	Two-stage surgical procedure	bladder stoma	Fever after surgery	Urethral operation history
Age	r	1.000	0.125	0.179*	0.630**	− 0.152	− 0.217*	0.110	0.236**	0.300**	0.280**	0.135	0.475**
	*P* value	.	0.174	0.050	0.000	0.098	0.017	0.232	0.009	0.001	0.002	0.141	0.000
Pyuria before surgery	r	0.125	1.000	0.150	0.140	−0.090	− 0.061	− 0.052	0.061	− 0.098	0.194*	0.205*	0.098
	*P* value	0.174	.	0.102	0.127	0.330	0.510	0.573	0.510	0.289	0.034	0.025	0.285
Urinary comorbidities before surgery	r	0.179*	0.150	1.000	0.105	−0.353**	− 0.113	0.110	0.113	0.058	0.033	−0.016	0.471**
	*P* value	0.050	0.102	.	0.253	0.000	0.220	0.230	0.220	0.528	0.724	0.865	0.000
Urethral defect length	r	0.630**	0.140	0.105	1.000	0.042	−0.298**	0.227*	0.301**	0.389**	0.334**	0.160	0.461**
	*P* value	0.000	0.127	0.253	.	0.650	0.001	0.013	0.001	0.000	0.000	0.081	0.000
Penile chordee	r	−.0152	−0.090	−0.353**	0.042	1.000	0.077	−0.051	− 0.143	0.195*	− 0.117	0.004	−0.293**
	*P* value	0.098	0.330	0.000	0.650	.	0.404	0.578	0.120	0.033	0.203	0.962	0.001
Urethral plate tubularization	r	−0.217*	− 0.061	−0.113	− 0.298**	0.077	1.000	− 0.231*	− 0.899**	0.059	−0.136	− 0.161	−0.204*
	*P* value	0.017	0.510	0.220	0.001	0.404	.	0.011	0.000	0.521	0.138	0.079	0.025
Free flap from oral cavity	r	0.110	−0.052	0.110	0.227*	− 0.051	−0.231*	1.000	−0.021	0.164	0.029	0.035	0.202*
	*P* value	0.232	0.573	0.230	0.013	0.578	0.011	.	0.820	0.074	0.752	0.705	0.027
Penis vascular pedicle flap	r	0.236**	0.061	0.113	0.301**	− 0.143	−0.899**	− 0.021	1.000	−0.059	0.136	0.161	0.204*
	*P* value	0.009	0.510	0.220	0.001	0.120	0.000	0.820	.	0.521	0.138	0.079	0.025
Two-stage surgical procedure	r	0.300**	−.0098	0.058	0.389**	0.195*	0.059	0.164	− 0.059	1.000	0.041	−0.089	0.183*
	*P* value	0.001	0.289	0.528	0.000	0.033	0.521	0.074	0.521	.	0.655	0.333	0.045
bladder stoma	r	0.280**	0.194*	0.033	0.334**	− 0.117	−0.136	0.029	0.136	0.041	1.000	0.097	0.219*
	*P* value	0.002	0.034	0.724	0.000	0.203	0.138	0.752	0.138	0.655	.	0.292	0.016
Fever after surgery	r	0.135	0.205*	− 0.016	0.160	0.004	−0.161	0.035	0.161	−0.089	0.097	1.000	0.202*
	*P* value	0.141	0.025	0.865	0.081	0.962	0.079	0.705	0.079	0.333	0.292	.	0.027
Urethral operation history	r	0.475**	0.098	0.471**	0.461**	− 0.293**	− 0.204*	0.202*	0.204*	0.183*	0.219*	0.202*	1.000
	*P* value	0.000	0.285	0.000	0.000	0.001	0.025	0.027	0.025	0.045	0.016	0.027	.

*P < 0.05

**P < 0.01

## Discussion

Hypospadias is the abnormal location of the urethra on the ventral surface of the penis with variable associations with the aborted development of the urethral spongiosum, ventral prepuce, and penile chordee [11]. Hypospadias surgery has been continuously evolving since its description by Celsius and Galen in the first and second centuries AD to improve suboptimal functional and cosmetic results. In spite of the advanced surgical techniques, the rates of complication after hypospadias repair remain high [12, 13]. One of the most common complications of hypospadias repair is urethrocutaneous fistula. Small-sized fistulas may disappear spontaneously, but most fistulas need surgical correction [14]. The incidence of urethrocutaneous fistula after hypospadias repair ranges from 6.20 to 38.8% [8, 9, 15–22], mostly during 10–20%. In the current study, the fistula rate was 32.5%, higher than the most studies. The possible reason was listed as below. Firstly, the most studies focused on the children while the enrolled patients in our study were defined as Tanner 4 or 5. Secondly, in our study, the rate of at least one operation was 51.67%, obviously higher than that in other studies, indicating that urethral operation history plays an important role in development of the urethrocutaneous fistula.

The success of the hypospadias repair can be attributed to good tissue and vascular supply [19], which may be associated with patient age and the number of operations patients have undergone before. The rate of hypospadias repair complications ranges from 10.1 to 37.5% in adult patients undergoing a primary repair but more than doubles to between 27.5 and 63.6% [23, 24] in patients with at least one urethral operation. Urethral plate is a healthy tissue with an extensive vascular network and muscle support [25]. Therefore, urethral plate is the ideal material for hypospadias repair. The higher complication rate in patients undergoing at least one urethral operation may be due to lack of healthy urethral plate, which should be healed by scarring instead of epithelization [26]. In addition, previously repair lead to subsequent distortion of anatomy and vasculature. Urethrocutaneous fistula in these patients was probably associated with poor tissue quality and tissue ischemia [19].

Few studies have focused on the correlation between urethrocutaneous fistula and urethral defect length. Huang et al. [20] revealed that urethrocutaneous fistula occurred in 8.2% (5/61) patients with urethral defect length less than 2 cm, 12.8% (9/70) cases with urethral defect length of 2–3 cm and 22.6% (7/31) with urethral defect length of 3–4 cm. However, the 5 patients with urethral defect length > 4 cm did not developed urethrocutaneous fistula after surgical repair. The relatively small number of patients with urethral defect length > 4 cm may be responsible for it. Yildiz et al. [21] indicated that patients with mid-penile hypospadias had a 1.7-fold increase in surgical complications compared to those with distal hypospadias (18.4% vs 10.4%) and 1.3-fold increase in fistula complications (7.8 vs 5.9%). Khan et al. reached the similar result [9]. The current study suggested that urethral defect length may be another independent risk factors of urethrocutaneous fistula. It is suspected that the longer defect length need better tissue and richer vascular supply to repair and healing of two different kinds of tissues was relatively difficult [20].

From several studies, age is the risk factor for hypospadias repair. Huang et al. concluded that older children (> 6 years) with hypospadias repair were more subjected to urethrocutaneous fistula. Yildiz et al. [21] reported the rate of urethrocutaneous fistula was significantly higher in those aged over 10 years. Several potential explanations might account for it. First of all, erection was taken into consideration as evidenced by research on adult patient with hypospadias [27]. With the increasing age, erection occurred more frequently, resulted in postoperative bleeding and dehiscence, and affected the postoperative complications notablely, especially for urethrocutaneous fistula [23]. Secondly, adolescent or adult hypospadias patients are much more likely to have undergone at least one urethral operation. In addition, adolescent or adult patients have different issues that may affect overall surgical success such as different skin and hair flora that may lead to perioperative infection [23]. Skin appendages, such as hair follicales, are potential microbial reservoirs [28]. Moreover, it is widely known that the healing ability of younger children is stronger than the older, which might be another reason for the lower incidence after successful surgical repair in younger patients [20]. In our study, patients with urethrocutaneous fistula were obviously older than patients without urethrocutaneous fistula. However, age was not the independent risk factor from the multivariate regression. Maybe prepuberty patients were excluded from our study and age was not so important among the patients defined as Tanner 4 or Tanner 5.

Despite the controversial status, a lot of the hypospadiologists favour the urinary diversion. From our study, urinary diversion after surgery had no significant difference between two groups.In addition, none of penile chordee, urinary comorbidities before operation, the pyuria before surgery, fever after surgery and surgical protocol had significant difference between two groups, which is different from the previous study [16]. It can be supposed our sample size is limited.

To sum up, our study also had some limitations. Because of the retrospective study, some parameters, such as surgical time, urethral plate width, glans size, urine culture result, GMS score and HOPE score evaluated before operation were not collected for analysis. GMS score could describe the severity of hypospadias with high inter-observer reliability [29] and have strong correlation with the risk of a surgical complication in the patients undergoing primary hypospadias repair [30]. Degree of chordee (S score) is independently prediction of fistula rate [30]. HOPE score was evaluated as an objective outcome measure of the cosmetic result after hypospadias surgery [31]. Furthermore, Confounding factors cannot completely excluded. In the further prospective study, the above limitation s will be taken into consideration.

## Conclusions

Urethral defect length and urethral operation history should be taken into consideration when planning hypospadias surgery since our study discovered that the risk of developing urethrocutaneous fistula after hypospadias repair is associated with urethral defect length and urethral operation history. Age, surgical procedure, type of surgical repair, chordee degree and other factors were not obviously related to the development of urethrocutaneous fistula.

## Additional file

Additional file 1: The data of the patients received hypospadias repair in the current research. (XLSX 15 kb)

## Abbreviations

AD: Anno Domini
OR: Odds ratio
SD: Standard deviation
